# Applying the principles of adaptive leadership to person‐centred care for people with complex care needs: Considerations for care providers, patients, caregivers and organizations

**DOI:** 10.1111/hex.13174

**Published:** 2020-12-19

**Authors:** Kerry Kuluski, Robert J. Reid, G. Ross Baker

**Affiliations:** ^1^ Institute for Better Health Trillium Health Partners Mississauga ON Canada; ^2^ Institute of Health Policy, Management and Evaluation Dalla Lana School of Public Health University of Toronto Toronto ON Canada

**Keywords:** adaptive leadership, chronic disease management, health services, Person‐centred care, quality improvement

## Abstract

**Background:**

Health systems in many countries see person‐centred care as a critical component of high‐quality care but many struggle to operationalize it in practice. We argue that models such as adaptive leadership can be a critical lever to support person‐centred care, particularly for people who have multiple complex care needs.

**Objective:**

To reflect on two concepts: person‐centred care and adaptive leadership and share how adaptive leadership can advance person‐centred care at the front‐line care delivery level and the organizational level.

**Findings:**

The defining feature of adaptive leadership is the separation of technical solutions (ie applying existing knowledge and techniques to problems) from adaptive solutions (ie requiring shifts in how people work together, not just what they do). Addressing adaptive challenges requires identifying key assumptions that may limit motivations for change and the behaviours influenced by these assumptions. Thus, effective care for patients, particularly those with multiple complex care needs, often entails helping care providers and patients to examine their relationships and behaviours not just identifying technical solutions. Addressing adaptive challenges also requires a supportive and enabling organizational context. We provide illustrative examples of how adaptive leadership principles can be applied at both the front line of care and the organization level in advancing person‐centred care delivery.

**Conclusions:**

Advancing person‐centred care at both the clinical and organizational levels requires a growth mindset, a willingness to try (and fail) and try again, comfort in being uncomfortable and a commitment to figure things out, in partnership, in iterative ways. Patients, caregivers, care providers and organizational leaders all need to be adaptive leaders in this endeavour.

## 1 BACKGROUND

Health systems in many countries see person‐centred care as a critical component of high‐quality care^1^ but many struggle to put these principles into practice.^2^, ^3^ A body of research has begun to operationalize person‐centred care by outlining the actions and activities that facilitate its achievement. Evidence from previous studies also underlines that achieving person‐centred care requires more than just executing a list of activities.^3^, ^4^, ^5^, ^6^ Therefore, we argue that such ‘how to’ guides on person centredness may be insufficient unless there is an accompanying shift in the mindset of those providing and those receiving care.^6^ Some scholars have looked to *adaptive leadership* as a critical lever to more effectively operationalize person‐centred care.^7^, ^8^, ^9^

The defining feature of adaptive leadership is the separation of technical solutions (ie applying existing knowledge and techniques to problems) from adaptive solutions (ie requiring shifts in how people work together, not just what they do). Addressing adaptive challenges requires identifying key assumptions that may limit motivations for change and the behaviours influenced by these assumptions. Thus, effective care for patients, particularly those with multiple complex care needs, may entail helping care providers and patients to examine their relationships and behaviours not just identifying technical solutions. This inference is supported by a growing body of research that speaks to the importance of trust and clear communication when supporting people with multiple complex care needs.^10^, ^11^, ^12^, ^13^, ^14^

In this paper, we concur with previous scholars who have described complex chronic care as an adaptive challenge^7^, ^15^ and argue that on‐going trial and error, learning from mistakes, attention to social context, a willingness to be vulnerable and a growth mindset are required to address the adaptive challenge. Adaptive leadership is required from all parties in care interactions (including patients, caregivers, care providers and organizational leaders).

This commentary is divided into three core sections. First, we briefly explore the concept of person‐centred care and suggest that the principles of adaptive leadership can help to meaningfully operationalize it. Second, we provide examples of how adaptive leadership principles can be translated to person‐centred care at the front‐line care level (by care providers, patients and caregivers) and the barriers that may be experienced. And, third, we explore how adaptive leadership principles can be translated to person‐centred care at the health‐care organizational level. We reflect on two bodies of literature and describe, through illustrative examples, how principles of adaptive leadership can help to guide the fundamental shifts that are required for person‐centred care delivery.

### 1.1 What are person‐centred care and adaptive leadership? How are these two concepts related?

Person‐centred care is a partnership between patients and their care providers through which the specific needs, preferences and expectations of patients and their caregivers (family and friends who provide care) are continually sought out, respected and considered in care planning, in the execution of care, and adaptation of care over time.^16^, ^17^, ^18^ Patient (or person)‐centred care, as a concept, can be traced back to the works of Carl Rogers^19^ and Michael Balint.^20^ Rogers, a psychologist, coined the related term *client‐centred therapy,* while Balint, a psychoanalyst, coined the term patient‐centred medicine. Both concepts articulate the person as more than the sum of their physical health conditions.^21^

Despite a growing interest in person‐centred care over the past 2 decades, rhetoric about person‐centred care has often moved ahead of practice because care systems remain rooted in a traditional model where *expert* care providers react to disease‐related issues that *passive* patients present.^22^, ^23^ Based on their expertise, care providers offer care options to people who then consent to these interventions or approaches. However, increasingly, people's care needs are focused on chronic conditions, which require a significant and active care role for patients and caregivers, particularly in the self‐management and monitoring of conditions between health system visits.^24^, ^25^ Chronic on‐going illnesses may be straightforward to treat and manage on their own, but become complex when they occur in combination (multimorbidity), or produce complex symptoms impacting physical and cognitive abilities or social factors (caregiver strain, diminished finances, unmet housing needs, etc). Such complex chronic illnesses are often challenging to treat and require time, trial and error, and on‐going management by a team of care providers including patients and their caregivers.^26^, ^27^ Effective management of chronic illness, particularly when multiple conditions co‐exist, requires a person‐centred approach: an understanding of both the conditions that people present with and their social context (networks, social supports, financial health, housing, nutritional needs, capacity to support their day‐to‐day activities)^26^, ^28^, ^29^; that directly influence people's abilities to access care, successfully self‐manage and achieve optimal health outcomes.^30^, ^31^ Most importantly, person‐centred care considers the views and preferences of patients and caregivers as the most critical inputs into care plans.^32^, ^33^ Strong and on‐going relationships between care providers, patients and caregivers are a fundamental component of person‐centred care. While some care providers (particularly, those in primary care medicine, nursing, geriatrics, social work, occupational therapy and physical therapy) provide care that considers both health and social care needs, the care experiences of patients, particularly within acute care‐oriented settings, often remain fragmented and focused on specific problems, not the overall health of individuals.

A number of authors have developed frameworks which seek to operationalize person‐centred care as a set of ‘action items’ or ‘activities’ that can be carried out by care providers, including but not limited to assessing patient and caregiver priorities, being honest and transparent, allowing more time during clinical appointments, and providing a point person for patients and families to follow‐up with when questions arise.^2^, ^3^, ^14^, ^34^, ^35^

However, implementing person‐centred care relies not only on individual skills, but also on supportive leadership that creates a practice environment enabling and sustaining these behaviours. Individuals occupying leadership positions in health care need to help to shift organizational norms and encourage staff and care providers in evolving their relationships with patients and caregivers. Such shifts in mindsets and relationships require *adaptive leadership skills,* that is a leadership mindset that assists people in tackling tough challenges and thriving in complex and challenging environments.^36^ Adaptive leadership enables organizational leaders and staff to develop competencies for supporting patient‐centred care: the knowledge, skills and behaviours critical to facilitate new actions and supports. While other leadership approaches, such as transformational leadership, situational leadership, distributed leadership^37^ and person‐centred leadership,^38^ may also enable major shifts in behaviours, roles and relationships, we focus here on adaptive leadership, whose core ideas seem most closely aligned to the changes necessary to move person‐centred care from mechanistic to meaningful.

Adaptive leadership is a framework developed over thirty years ago by Ron Heifetz and Martin Linsky at Harvard.^36^ Heifitz and Linsky identified a critical issue that undermined leaders’ efforts to introduce transformational change: the failure to recognize adaptive problems and approach them differently from *technical* problems.^39^ A technical problem can be managed with technological fixes or programmatic solutions. A technical problem in health care could be the introduction of new methods for repairing a broken hip or replacing a faulty heart valve. These problems can be challenging and complex, but build on the experience and underlying knowledge of the care providers involved. An adaptive challenge is different in that it may have many inputs and require changes in the ways of thinking and doing that cannot be solved by improving technical expertise alone. These solutions must start with an examination of current priorities, assumptions, habits and loyalties. Technical solutions can be complicated, but adaptive challenges are more difficult since there are rarely clear immediate solutions. Instead, the approaches to adaptive challenges rely on thoughtful reflections on what to preserve from past practices, what to discard, and how to create new approaches that build from the best of the past.^39^ Heifetz et al^39^ emphasize that a common mistake for leaders (specifically care providers, as illustrated in our example below) is applying technical solutions to adaptive challenges. For example, a care provider designing a self‐care regimen for a patient with complex care needs may fail unless they consider the social factors that influence illness management (such as a patient's ability to purchase medications, arrange transportation to get to appointments and secure meals and nutritional support). A technical solution, like a prescription, will not yield the intended results unless these other adaptive challenges are considered. Through trial and error, patients, caregivers and care providers can learn how to address these adaptive challenges over time by testing some approaches, seeing what works and making adjustments along the way. Organizational leaders also have to differentiate between technical solutions and adaptive challenges. For example, while there may be straightforward approaches for some challenges (eg hiring more staff) adaptive challenges (eg addressing low satisfaction and poor experiences among patients and families) may require examining provider, patient and caregiver behaviours, attitudes and assumptions and by creating a safe space to articulate concerns and ideas for improvement.

The CODE concept^40^ (**C**haracter, **O**rganizational justice, **D**evelopment and **E**motional intelligence) may be helpful in supporting care providers and organizational leaders in understanding and enacting the key tenets of adaptive leadership. CODE dimensions include character (owning your mistakes and building trust); organizational justice (ensuring open communication); development (being willing to explore new ways of doing things); and emotional intelligence (recognizing the realities and needs of others/showing empathy). The CODE concept addresses several core tenets of person‐centred care and emphasizes vulnerability and adaptability.

Person‐centred care requires strong relationships between care providers and organizational leaders *and* between patients and caregivers to enable adaptive work. Not surprisingly, a key challenge is that many care providers, patients and caregivers may not be open to stepping back, reflecting on the challenges and committing to approaching their problems in new ways. The willingness and ability to engage in adaptive work has been termed *adaptive capacity*.^39^ In the next section, we explore how adaptive leadership can be translated to person‐centred care at the front‐line clinical care level. The examples illustrate how care providers can enhance their adaptive capacity and understand the factors that may shape patients’ and caregivers’ adaptive capacity.

### 1.2 How can adaptive leadership principles translate to person‐centred care at the front‐line clinical care level?

Bailey et al ^8^ and Heifetz et al^39^ note that leadership includes actions by front‐line care providers, patients and caregivers. Expert clinicians who prescribe a new treatment or self‐management regimen for a patient may be unsuccessful in helping their patients achieve desired outcomes unless they take the time to understand their patients’ needs, co‐develop a workable plan and inspire change through enabling, ongoing and trusting relationships. Leadership in these relationships requires collaboration, a willingness to embrace uncertainty, to try things differently and to adjust as needed. Creating the adaptive capacity of an individual or group requires meaningful conversations to inspire change. Quirk et al^41^ discuss three kinds of leadership conversations: adaptive, alignment and courageous. In addition, Heifetz et al^39^ identify the key behaviours that enable adaptive leadership and change, as the iterative process of *observing, interpreting and intervening*. In Table 1, we define each of these concepts and describe how they translate to person‐centred care at the clinical care level.

**Table 1:** How adaptive leadership concepts translate to person‐centred care

**Adaptive Leadership Concept**	**Definition**	**How it Translates to Person‐Centred Care**
Adaptive Conversation	Inspiring leadership in others by helping them to reflect on challenges, think ahead, and plan for the future.	Reviewing goals of care and priorities including how to prepare for the future and what to expect in the disease trajectory; patients, caregivers and care providers manage expectations of each other.
Alignment Conversation	Identifying and discussing the underlying reasons for people's resistance to change and providing a safe space to talk about concerns.	Explicitly asking about fears and concerns, demonstrating compassion and empathy when communicating.
Courageous Conversation	Correcting unacceptable behaviours or respectfully calling out a discrepancy in others’ behaviours.	Care providers being honest about the likelihood of a poor outcome due to a patient's behaviours. At the same time, patients and caregivers verbalizing their discomfort when they feel their preferences are not being considered. Patients and caregivers need to feel safe in speaking out without fear of reprisal.
Observing	Heifetz and Linsky^39^ use the analogy of ‘getting off the dance floor and onto the balcony’. From the balcony, you can see the broader context or the ‘big picture’ which can inform a greater understanding of issues and actions.	For care providers, it is about understanding the social context of patients and caregivers to identify factors that will influence their ability or willingness to manage their conditions. For patients and caregivers, it is about recognizing care providers’ constraints in their ability to support them (such as a lack of time or resources, high patient demand, lack of evidence base of suitable treatments). Like any relationship, acknowledging the constraints of the other party is critical in creating a sustainable, respectful relationship and preventing burn‐out.
Interpreting	Reading between the lines and not taking everything at face value. Heifetz et al^39^ describes *interpreting* metaphorically as ‘*listening for the song beneath the words’*.	Paying attention to body language, facial expressions and what is not being said. Such intentional listening requires patience, time, trust, probing and comfortable silence. Continuity of care between the care providers, patient and caregiver is required.
Intervening	Reflecting on the hypothesis of the problem. Any proposed ‘intervention/solution’ should be considered a ‘trial’ which may need to be adapted over time. The ‘intervention’ should be clearly connected to a shared purpose and take into account the resources available.	Trying a new treatment or care plan that reflects the shared goals of the provider(s), patient and caregivers with the caveat that things may need to be tweaked and changed over time (continually testing what works and what does not work). The new treatment/care plan needs to leverage available resources of the patient/caregiver (including their access to financial resources and caregiver capacity). It is important here that a balance be struck between giving a treatment or plan enough time to succeed versus pivoting to a new strategy too quickly.^40^

To enable adaptive change, care providers, patients and caregivers should engage in the types of adaptive conversations described in Table 1, which promote the potential for shared leadership and decision‐making as opposed to a conventional command and control approach.^41^

Several authors have linked adaptive leadership to person‐centred care in front‐line clinical care. Thygeson et al^9^ emphasize that misapplying technical solutions to adaptive challenges could lead to harm, a false sense of progress and immense frustration for care providers. They underscore the potential of adaptive strategies to facilitate the ‘patients’ adaptive health work’ (p.1009) in the practice of medicine, and they emphasize the clinician's role in leading patients through this adaptive change.

Drawing similar conclusions, Anderson et al^7^ developed the Adaptive Leadership Framework for Chronic Illness. They suggest that adaptive approaches are effective in managing chronic illness, given the uncertain and changing patient trajectories that often characterize longer‐term conditions. They underline the importance of care providers’ understanding the adaptive capacity of patients in order to best support them and develop a workable plan.

Tait et al examined adaptive practices among heart failure teams. How care providers engaged with patients and caregivers was influenced by many factors including patient complexity, the provider's perspective on complexity (did they recognize the need to respond differently?) as well as the quality and strength of relationships between the providers and patients.^42^

None of these examples emphasizes the need for primary ‘informal’ caregivers to develop adaptive capacity to deal with the stress and load associated with caring for people with complex care needs. The success of an intervention or treatment plan for a patient may fail without consideration of the caregiver's capacity. Nimmon et al^43^ identify the importance of understanding the changing nature of the patient‐caregiver dyad in heart failure management, particularly the needs of the caregiver, who may also be vulnerable and experiencing illness. This dynamic will influence adherence and outcomes and could be missed by care providers if they are solely focused on the patient.

In engaging patients *and* caregivers in adaptive change care providers must consider the barriers they may face in doing so. Carman et al^44^ identify factors that influence patient engagement at the individual level (their beliefs about their role, prior health‐care experiences, their functional capacity, health literacy, etc); the organization level (policies, practices and culture); and the societal level (social norms, regulations and policies that create conditions and norms for citizen participation). These factors may help us understand w*hy some patients and caregivers wish to play an active role in their care while others do not*. Patients and caregivers will have different preferences when engaging in care planning and decision making, and this may change for several reasons, including the nature of their illness, the type of decision in front of them and their relationships with their care providers.^45^, ^46^, ^47^, ^48^

In addition to the adaptive leadership skills needed by care providers to guide patients and caregivers through change, care providers face their own adaptive challenges. Care providers may face challenges in stepping outside their roles as experts to work in teams, across care boundaries and to be vulnerable in accepting the discomfort and ambiguity felt by patients and caregivers. Incentives, workplace expectations of efficiency and using evidence‐guided recommendations may create barriers to exercising person‐centred care and adaptive leadership, leading care providers to feel like they are constantly reconciling competing pressures. Care providers may also experience roadblocks when they lack access to needed resources due to stringent eligibility criteria, funding limitations and other policy barriers.^43^ Individual providers can find the tensions of balancing their efforts to provide person‐centred care and growing workplace pressure difficult to reconcile on their own.

### 1.3 How can adaptive leadership principles support person‐centred care at the health‐care organizational level?

Organizational leaders play an important role in helping care providers, patients and caregivers to address the adaptive challenges of complex chronic care and overcome the barriers described in the previous section. While individual skills are critical for adaptive leadership behaviours, their development is supported by a culture that recognizes and rewards such behaviours. Care providers are more likely to support patients and caregivers in developing adaptive capacity if they practice within *engagement capable environments (ECE)*.^49^, ^50^, ^51^ In ECEs, organizational leaders believe in (and actively support) the building of adaptive capacity of care providers through explicit articulation of values and practices that acknowledge and actively support their needs.^49^, ^50^, ^51^ One example of what this can look like is provided by Corazzani et al^52^ in their paper, ‘Implementing Culture Change in Nursing Homes: An Adaptive Leadership Framework’. The authors describe adaptive challenges and the leadership behaviours needed to support culture change in the long‐term care sector. Through a series of focus groups with nursing staff, medical care providers and administrators in nursing homes in the United States they explored barriers and facilitators to culture change. In their analysis, they identified adaptive challenges along with strategies to address these challenges (which they refer to as adaptive leadership responses). An example of an adaptive challenge was that the insights of nursing assistants regarding the residents for whom they cared were not being incorporated into care decisions. Several adaptive leadership responses were identified including managers providing care to residents during care planning meetings so nursing assistants could attend and provide input. This response enabled nursing assistants to introduce the perspectives of residents which is particularly important when residents are unable to articulate their needs. Such deliberate activity is an example of how environments can be made more *engagement capable*.

The importance of engagement capable environments is also illustrated in O’Connor et al's paper ‘The Leadership and Organizational Context Required to Support Patient Partnerships’. ^53^ In a series of case examples, their paper illustrates the importance of distributed leadership and shared decision making at the organizational and front‐line levels among organizational leaders, staff, patients and families. For example, starting in 2011 the Huron Perth Healthcare Alliance in Ontario, Canada, developed unit‐level action councils with patient partners; this was followed by open visitor policies, use of bedside whiteboards to improve communication with families, patient experience councils, standard uniforms for care providers so they could be easily identified by patients, and (most recently) bedside end of shift reporting with families so they could be included in care planning. These practice changes, were directly informed by the suggestions of patients and families, enabling patients and families to be partners in care planning, and by extension, adaptive leaders in their care. The Huron Perth example also demonstrates how momentum for patient engagement activities can build over time.

Examples from related literature on situational leadership also underline the importance of leadership behaviours in creating conditions that support patient‐centred care. Rokstad et al, in their study of three nursing homes^54^ in Norway found that when leaders in nursing homes had a long‐term focus on professional development, were regularly present on the wards and knew the skills and needs of front‐line staff, staff felt more supported and engaged. The dementia care mapping (DCM) experience that was conducted in this particular study also provides an example of what person‐centred care can look like for people with cognitive impairment. DCM^55^, ^56^ involves observations conducted by a trained professional of the interactions between staff and residents which are documented and shared with staff in a feedback session. Staff then redesign care plans and identify changes in how staff might interact with dementia patients.

In summary, to execute the principles of person centredness, providers, patients and families require the mindset and tools to be adaptive leaders. We need to acknowledge the complex systems in which they work as well the challenges that they face. Such realities require care providers, patients, caregivers and leaders to ‘think outside the box’ within environments that allow them to creatively explore solutions, acknowledge and learn from mistakes and continually strive to improve.

## 2 CONCLUSION

We emphasized in our paper that applying principles of person‐centred care should not be done mechanistically (eg following a checklist), but rather, that person‐centred care may be authentically realized by applying the principles of adaptive leadership. Advancing person‐centred care at both the clinical and organizational levels requires a growth mindset, a willingness to try (sometimes fail) and try again, comfort in being uncomfortable and a commitment to figure things out, in partnership, in iterative ways. Patients, caregivers and care providers are all adaptive leaders in this endeavour. It all starts with openly acknowledging challenges and uncertainty (embracing vulnerability), letting everyone have a voice and being willing to try new things. As stated by Brené Brown, ‘*vulnerability is not weakness […] it is the birthplace of innovation, creativity and change’*.

## 3 LIMITATIONS

This paper is a commentary that explores and combines two concepts relevant to the advancement of quality in health systems: person‐centred care and adaptive leadership. We did not conduct a systematic or scoping review of the literature, though we did conduct a rapid scan of articles that have combined person‐centred care with adaptive leadership in order to provide illustrative examples in our commentary. This paper therefore is not an exhaustive or systematic account of the historical origins and uses of these concepts. Given the challenges in advancing person‐centred care in a meaningful way, our paper provides one frame for thinking about how to meaningfully advance person‐centred care in practice.

## 4 CONFLICT OF INTEREST STATEMENT

The authors declare no conflict of interest.
